# Sleep and Health

**Published:** 1891-08

**Authors:** 


### HALL'S JOURNAL OF HEALTH

TRUTH DEMANDS NO SACRIFICE; ERROR CAN MAKE NONE.

Vol. 38. AUGUST, 1891. No. 8.

### SLEEP AND HEALTH.

We have been told, time after time, that early rising is a wonderful method of lengthening the day, and thus adding to the value and usefulness of a man's life. To those who are tempted to self-indulgence in sleep by a natural tendency to ease, or a lack of incentive to early rising, the injunction is good enough, and should be enforced. But with the majority of us the danger lies in the opposite direction. The demands upon our time are so many and so pressing, that the longest day is sadly too short for the work we would like to see done in it. We go to bed at night with the unpleasant conviction that something is being left half finished, and to-morrow has already enough duties of its own to occupy its hours. So we get into the habit of scheming how the day may be made a bit longer by stealing a fragment from both ends of the night. We then flatter ourselves upon having stolen a march upon Dame Nature and cheated her out of her own. And how easy it is to imagine that we feel none the worse for the change, and that the touch of weariness which sometimes oppresses us is an unworthy inclination to slothfulness that ought to be kept in subjection.

Time, however, tells another tale, and a few years will prove that the human system must have the appointed hours of sleep and rest, or serious consequences will follow. When early rising is practised it must be preceded by early retiring. We are told that a large proportion of the people who have lived to an extreme old age were early risers ; but it is well to understand that they are mostly agricultural laborers, and country people, living simply and contentedly, and going to bed about nine o'clock in the evening, without a care to ruffle their sleep. A school boy on his holiday does not rest more soundly ; and it is unreasonable for the ordinary business man, who has hands and brain actively engaged until almost midnight, to attempt keeping pace with him in the morning. There is no real gain in robbing one's self of the needed rest. The day may be lengthened but it is proportionately weakened, for loss of sleep means loss of energy.

There is a story told of a certain tradesman who was in difficulties, and went to his rich brother for assistance. On his arrival he found him in bed, and had to wait some time for his appearance. "I am surprised at you staying in bed so long," said the poor relation ; "I have been up three hours at least." "Yes," replied the more fortunate brother, "but you see when I do get up I am thoroughly awake." The hint was more forcible than thoughtful, yet it contains a lesson which is especially applicable to those who are trying to gain for themselves a livelihood and a fortune. He who has enough sleep has secured one of the safeguards against the encroachments of disease and mental prostration. His nerves are steadier, his intellect is clearer and keener, and the business and responsibility of life are attended to with a degree of comfort and efficiency that are not otherwise attainable.

A few days ago, two gentlemen met by chance in our sanctum. They were quite youthful in appearance, and in excellent health. On comparing notes it was found that both had been in the military service during the "late unpleasantness," but upon opposite sides. Their youthful appearance led to a banter as to which was the elder, and it was agreed that each should mark the date of his birth on a slip of paper, and place it in the writer's hands, when it transpired that the "wearer of the gray" was senior by some seven years. We confessed our astonishment, for he was by far the younger looking. "I am a great home-body," he explained, "and having married soon after the war, I have scarcely ever missed retiring for the night as early as nine o'clock. In addition to this, my habits of life have always been simple and temperate."

Here was a man, above fifty years of age, who did not look to be more than thirty-five, all owing to simple and regular habits of life, with a due allotment to "nature's sweet restorer, sleep." And nature will never be cheated of her requirements, without writing her protest in indelible lines.

